# Fabrication, characterization and numerical validation of a novel thin-wall hydrogel vessel model for cardiovascular research based on a patient-specific stenotic carotid artery bifurcation

**DOI:** 10.1038/s41598-024-66777-5

**Published:** 2024-07-15

**Authors:** Ashkan Shiravand, Kevin Richter, Pia Willmann, Pepe Eulzer, Kai Lawonn, Anna Hundertmark, Giorgio Cattaneo

**Affiliations:** 1 Institute of Biomedical Engineering, University of Stuttgart, Stuttgart, Germany; 2 Faculty of Natural and Environmental Sciences, University of Kaiserslautern-Landau, Landau, Germany; 3 Faculty of Mathematics and Computer Science, University of Jena, Jena, Germany

**Keywords:** Cardiovascular engineering, Hydrogel, Fluid dynamics, Vessel compliance, Vessel model, In vitro, Numerical simulation, Fluid–structure interaction (FSI), Ultrasound, Biomedical engineering, Vascular diseases

## Abstract

In vitro vascular models, primarily made of silicone, have been utilized for decades for studying hemodynamics and supporting the development of implants for catheter-based treatments of diseases such as stenoses and aneurysms. Hydrogels have emerged as prominent materials in tissue-engineering applications, offering distinct advantages over silicone models for fabricating vascular models owing to their viscoelasticity, low friction, and tunable mechanical properties. Our study evaluated the feasibility of fabricating thin-wall, anatomical vessel models made of polyvinyl alcohol hydrogel (PVA-H) based on a patient-specific carotid artery bifurcation using a combination of 3D printing and molding technologies. The model’s geometry, elastic modulus, volumetric compliance, and diameter distensibility were characterized experimentally and numerically simulated. Moreover, a comparison with silicone models with the same anatomy was performed. A PVA-H vessel model was integrated into a mock circulatory loop for a preliminary ultrasound-based assessment of fluid dynamics. The vascular model's geometry was successfully replicated, and the elastic moduli amounted to 0.31 ± 0.007 MPa and 0.29 ± 0.007 MPa for PVA-H and silicone, respectively. Both materials exhibited nearly identical volumetric compliance (0.346 and 0.342% mmHg^−1^), which was higher compared to numerical simulation (0.248 and 0.290% mmHg^−1^). The diameter distensibility ranged from 0.09 to 0.20% mmHg^−1^ in the experiments and between 0.10 and 0.18% mmHg^−1^ in the numerical model at different positions along the vessel model, highlighting the influence of vessel geometry on local deformation. In conclusion, our study presents a method and provides insights into the manufacturing and mechanical characterization of hydrogel-based thin-wall vessel models, potentially allowing for a combination of fluid dynamics and tissue engineering studies in future cardio- and neurovascular research.

## Introduction

According to the World Health Organization (WHO), cardiovascular diseases are the primary cause of mortality worldwide. Atherosclerosis, characterized by the buildup of fat and fibrous plaques in arteries, is the leading cause of heart disease and stroke^1^. The internal carotid artery (ICA) and external carotid artery (ECA), originating from the common carotid artery (CCA) at carotid bifurcation, supply blood to the neuronal structures of the brain and to the facial and neck tissues, respectively^2^. Local hemodynamic variables such as non-physiological and oscillating wall shear stress (WSS), characterized by complex fluid dynamics pathways, are substantial risk factors for the development and progression of atherosclerosis at carotid bifurcation and associated ICA stenosis (ICAs)^3^. Moreover, elevated shear stresses and turbulence adjacent or distal to stenotic regions heighten the risk of platelet activation and thrombus formation^4^, posing a significant risk of embolic ischemic stroke with substantial mortality and disability^5^. In the efforts to stabilize vessel wall and prevent embolism, endovascular stents may be implanted at the region of atherosclerotic carotid bifurcation. However, this intervention might inadvertently trigger in-stent thrombosis at the mechanical and chemical interface with the endothelium^6^.

To enhance treatment strategies and effectively prevent strokes, a deeper comprehension of the complex hemodynamics and the impact of endovascular interventions on fluid dynamics at carotid bifurcation is essential. In vivo studies using ultrasound have been performed to analyze the hemodynamics at carotid bifurcation, among other regions^7^. In vitro and in silico models of carotid arteries provide valuable insights into fluid dynamics and its correlation with artery disease development and progression observed in clinical imaging^8–11^. These models offer more reproducible and controllable test conditions with often less cost compared to in vivo studies and play a vital role in preliminary experimentation before proceeding to in vivo tests. In particular, in vitro^12,13^ and in silico^14^ models can potentially provide insights into the mechanisms of thrombus formation within stenotic regions and those triggered by implanted devices. Moreover, in vitro studies have highlighted the influence of vessel pulsation on endovascular fluid dynamics^15,16^ which is also likely to affect implant stability and its apposition to the vessel wall.

While previous studies have focused primarily on silicone-based block models lacking compliance characterization^14,17,18^ or standardized geometries^10^, Corbett et al.^19^ fabricated standardized aortic models with a 2-mm uniform thickness made of silicone using the an injection molding technique. The mold was manufactured by a CNC machine and the vessel models were characterized in terms of stiffness and local distensibility, whereas the best-fitted strain energy function to the experimental tensile data was used for the numerical simulation.

Given the widespread application of hydrogels in tissue engineering approaches and their potential in the endothelialization of vascular models^20–23^, in this preliminary study, we evaluated the fabrication and characterization of anatomical, thin-wall, and thus compliant patient-specific vessel models made from PVA-H using a combined 3D printing and molding technology. The vessel geometry was derived from patient-specific carotid artery bifurcation anatomy. Experimental data from the material tensile tests were implemented in a numerical model applying a linear elastic assumption with constant as well as strain-dependent elastic moduli for both PVA-H and silicone. The simulated volumetric compliance and local distensibility were compared with the results obtained from physical models. Finally, a feasibility study was performed to assess fluid dynamics within PVA-H carotid bifurcation model integrated into a mock circulatory loop.

## Methods

### Ethics approval and consent to participate

The principles of the Declaration of Helsinki and the requirements of medical ethics and confidentiality have been respected in this study. Only non-identifiable, retrospective CTA scan has been processed for the joint experimental and computational geometry. The German General Data Protection Regulation does not concern the processing of anonymous information, including statistical or research purposes, see https://dsgvo-gesetz.de/erwaegungsgruende/nr-26/.

### Anatomical data

**Figure 1:**
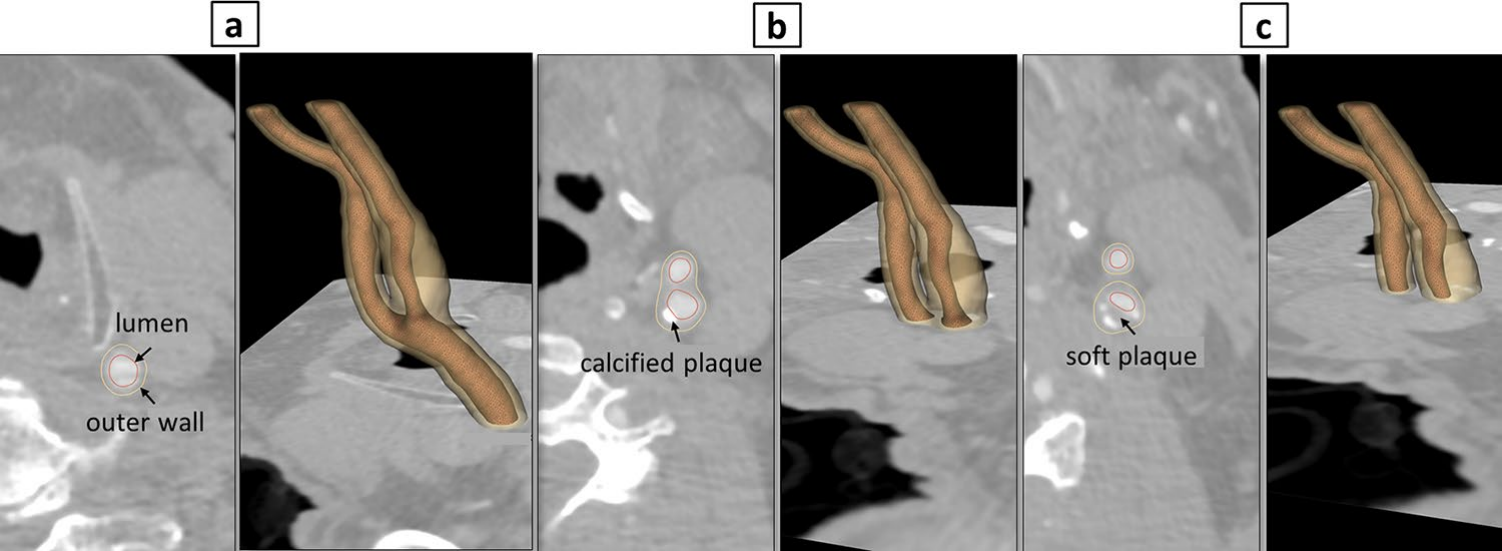
CTA slices with 3D projection of reconstructed carotid artery bifurcation: lumen and outer wall at CCA (**a**), bright spot showing calcified plaque at ICAs region (**b**), soft plaque seen between the calcified plaque and lumen (**c**).

The inner lumen and vessel wall geometries of the CCA and its branches at carotid bifurcation, encompassing the ICA and ECA, were derived from computed tomography angiography (CTA) scans of the head and neck region of a clinical patient (Fig. 1a). Eulzer et al. introduced and detailed the methodology for reconstructing the complete lumen domain from CTA slices, including the geometry and workflow^24^. For creating the vessel wall geometry in this study, since the outer wall was not visible in all CTA slices, the inner wall label map was initially inflated by 2 × 2 × 2 voxels, corresponding to an inflation thickness of 1.4 mm in each direction. Subsequently, the resulting outer wall was manually adjusted to match the slices where the outer wall label map was visible. The minimum inflation thickness of 1.4 mm aimed to reduce the risk of fissures during the molding process of the model while remaining within the observed range of carotid wall thickness in patients (0.5–1.53 mm)^25^.

Given that plaque develops within the arterial wall, the outer wall was assumed to encompass both soft and calcified plaques. Therefore, in regions with calcified plaques prominently visible on CTA images, the inner wall was manually inflated to encompass both types of plaques (Fig. 1b,c). Finally, the inner lumen and wall geometries of CCA, ICA and ECA were converted to STL file format for the design of the mold, comprising a core and a shell, as well as for geometrical characterization. Image processing, segmentation, model inflation, and extraction were performed using the open source software 3D Slicer^26^.

### Core and shell design

The vessel models were fabricated utilizing a mold composed of a core and two shell halves. Initially, the STL files of the carotid artery, comprising the vessel lumen and wall geometries of CCA, ICA, and ECA, were imported as “solid bodies” into Siemens NX 11.0 (Siemens Digital Industries Software, Texas, USA). The “solid body” representing the vessel lumen was used as the core and segmented into three branches: CCA, ICA, and ECA, in the Siemens NX. A separation plane, aligned with the vessel centerline, was created to bisect the mold into two shell halves, ensuring equal division of the ECA, ICA and CCA without undercuts. The “solid body” representing the wall geometry was merged with the vessel lumen, and the resulting merged body was subsequently subtracted from the shell halves.

**Figure 2:**
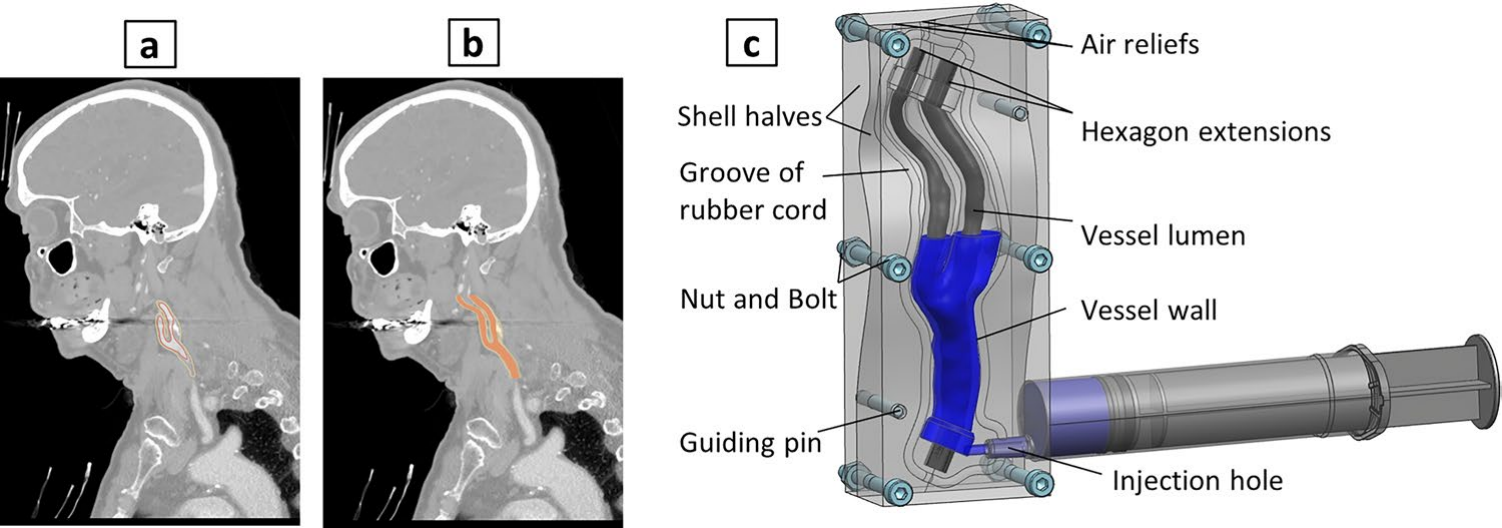
2D image cut (**a**) and projection (**b**) of the reconstructed carotid artery bifurcation on CTA sagittal slice of patient. 3D model depicting material injection into the core–shell mold of the carotid vessel (**c**).

Six holes were designed to fasten the shell halves together using six M3 nut-bolt pairs. Moreover, two holes for guiding pins were incorporated to facilitate the alignment of the shell halves. Hexagon profiles were appended to the ends of the core and extended by 10 mm to enable the fixation and alignment of the core lumen within the shell. A 1-mm diameter rubber cord was placed in the designed grooves between the shell halves to serve as a sealant. Furthermore, the design included a 4-mm diameter hole for polymer injection and two holes for vent relief purposes (Fig. 2).

Four planes in distinct vessel cross-sections—CCA, ICA, ICA, ICAs, and ECA—were defined and marked to enable subsequent comparisons of geometry and diameter distensibility between the fabricated and numerical models. The branches of five vessel cores were printed out of "Grey" resin using a stereolithography 3D printer (Form 3, Formlabs Inc., MA, USA). Additionally, five molds were printed out of "Clear" material using the same 3D printer. The two shell halves were aligned using guiding pins and fastened with nuts and bolts. Following the injection of the polymer and subsequent curing, the two shell halves were opened and the core was extracted.

### Material preparation and processing

The vessel models were fabricated using PVA-H and silicone (Dragon Skin 10 NV, Smooth-On Inc., PA, USA). For the preparation of PVA-H models, dimethyl sulfoxide (DMSO) (Merck KGaA, Darmstadt, Germany) and distilled water were mixed at 80 wt% and 20 wt%, respectively, and heated to 60 °C. Polyvinyl alcohol (PVA) powder (Merck KGaA, Darmstadt, Germany) with a molecular weight of 85,000–124,000 g/mol and 99 + % hydrolysis was added to the DMSO-water solution at 17 wt%. The mixture was stirred and heated at 120 °C until the PVA powder completely dissolved, yielding a homogenous solution. The resulting hydrogel was injected into the mold using a syringe through the gap between the shells and the core, resulting in the formation of a homogenous vessel wall geometry. The wall geometry integrated soft and calcified areas within stenotic region, all from the same material and thus without variation in mechanical properties. Slow injection was employed to minimize air bubble incorporation, resulting in a filling time of 40–50 s. The mold was then placed in a freezer at − 22 °C for 18 h, followed by thawing at room temperature for 6 h. The freeze–thaw cycle was repeated three times to ensure the proper gel formation.

The Dragon skin 10NV silicone consists of two components, parts A and B, mixed in a 1:1 ratio. The mixed silicone was subjected to vacuum degassing for 5 min to eliminate bubbles and subsequently cured at room temperature for 90 min. The prepared silicone was then injected into the molds using the same procedure described for PVA-H.

In total, five silicone and four PVA-H vessel specimens were successfully fabricated following demolding and core removal. The geometry of each vessel model was evaluated for comparison with extracted imaging data, volumetric compliance, and local distensibility. The PVA-H models were examined immediately after the final thawing cycle to minimize the gradual water loss through evaporation.

### Geometrical characterization

**Figure 3:**
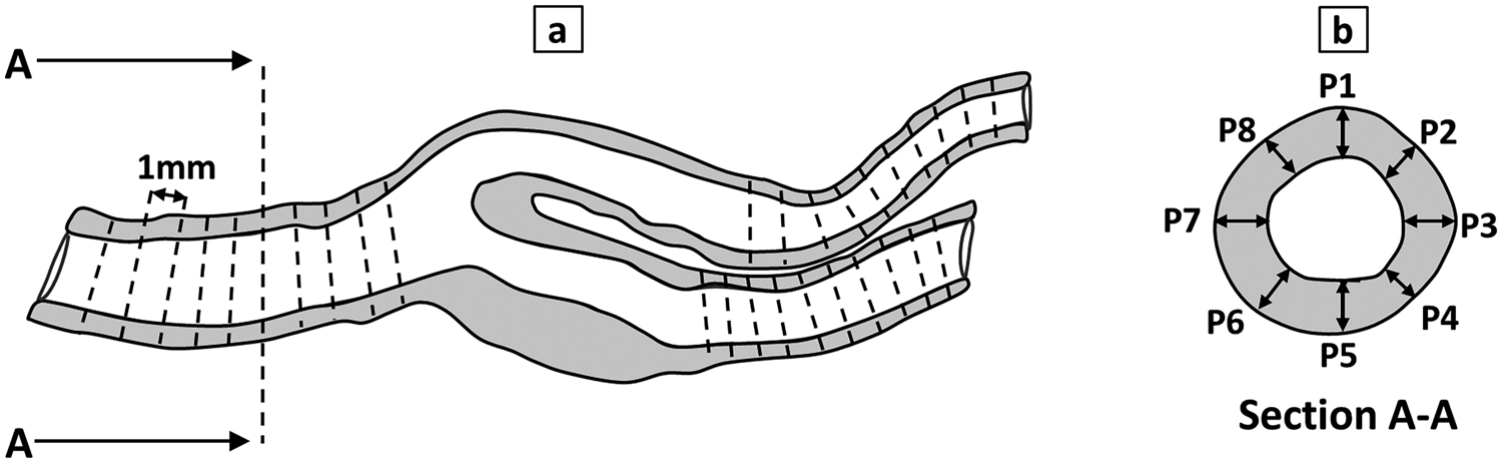
Schematic view section of the carotid artery bifurcation with equally-spaced slices at a distance of 1 mm (**a**) and eight circumferentially-spaced measurement positions (P1–P8) (**b**) for the measurement of wall thickness at the corresponding position.

The produced vessel models were cut into approximately 1 mm-thick slices along the vessel lumen centerline using a scalpel. Subsequently, the wall thickness was quantified using Leica S9D stereo microscope (Leica Mikrosysteme Vertrieb GmbH, Wetzlar, Germany) equipped with a camera (MikroCam II, Bresser GmbH, Rhede, Germany) at eight circumferentially spaced positions (P1–P8) across ten slices for CCA, ICA, and ECA, excluding ICAs, as depicted in Fig. 3. Additionally, following the conversion of the imaging data to STL models representing the inner lumen and wall geometries of the carotid bifurcation, these models were imported into Siemens NX as “solid body”. The wall thickness was then determined within the solid body of the wall geometry, following a similar procedure at eight circumferentially spaced positions (P1–P8) across the same ten slices for each branch, facilitating comparison with the fabricated vessel models.

Moreover, the perimeter of the vessel lumen was measured, and the effective diameter was calculated as D_eff_ = perimeter/π at each marked slice before comparison with the imaging data.

### Tensile stress–strain testing

To assess the stress–strain relationship, five cylindrical specimens of both materials were fabricated, each possessing a diameter of 8 mm (D = 8 mm) and a length of 100 mm (L = 100 mm). The stress–strain behavior of all the specimens was evaluated by a quasistatic tensile test, conducted at a test speed of 1 mm/min, utilizing a universal tensile testing machine 34SC-1 (Instron, Norwood, United States). The elastic modulus E was determined by fitting a linear trendline to the stress–strain curve up to 20% strain, covering the physiological strain range in arteries.

The elastic modulus was then implemented in the numerical simulation setup for the solid domain depending on the strain metric ${\varepsilon}_{\text{m}}$, which considers both volumetric and shear strains, i.e., $\text{E = E}({\varepsilon}_{\text{m}})$. The five experimental tensile tests conducted on PVA-H and silicone were averaged, and difference quotients representing the piecewise slopes of the averaged stress–strain curve were computed at specific strain values to represent values of strain-dependent modulus E $({\varepsilon}_{\text{m}})$. To obtain discrete modulus values, the strain was increased in 0.2% and 5% steps within the strain ranges of 0–10% and 10–100%, respectively.

### Volumetric compliance

**Figure 4:**
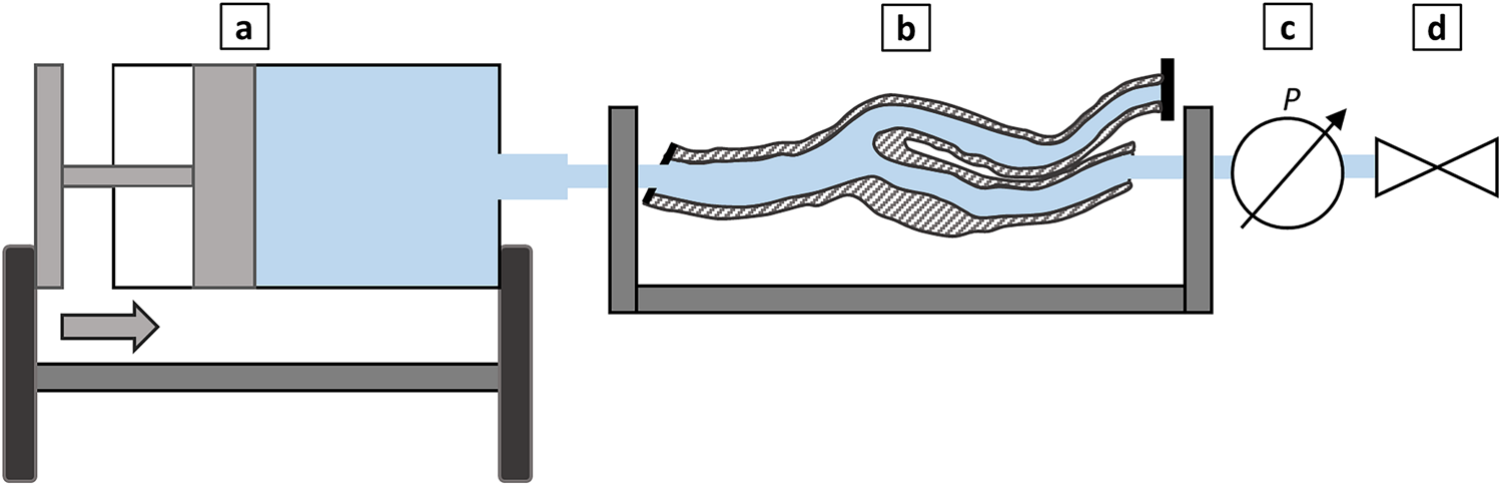
Schematic illustration of the compliance measurement setup filled with water: pump dispenser (**a**), vessel model (**b**), pressure transducer (**c**), and valve (**d**).

Arterial compliance was measured after connecting the vessel model to a syringe pump (Microlab m, Hamilton Bonaduz AG, Bonaduz, Switzerland) and a disposable pressure transducer (Combitrans, B. Braun Melsungen AG, Melsungen, Germany), as illustrated schematically in Fig. 4. The pump dispenser and the vessel model were filled with distilled water, and bubbles were removed before closing the valve. While the proximal end of the vessel was tightly connected to the syringe pump, the distal ends of the vessel were tightly sealed to maintain the defined volume necessary for compliance measurement. Upon valve in systolic (${\text{V}}_{\text{s}}$) and diastolic (${\text{V}}_{\text{d}}$) volumes by the differences in systolic (${\text{P}}_{\text{s}}$) and diastolic (${\text{P}}_{\text{d}}$) closure, additional distilled water was incrementally injected into the vessel model in 1-mm^3^ volume steps. The resulting increase was recorded after each step, and the volume-pressure curve was plotted. The compliance value was determined by dividing the differences pressures:

1 $$\text{C} = \frac{\Delta \text{V}}{\Delta \text{p}}\text{=}\frac{{\text{V}}_{\text{s}}-{\text{V}}_{\text{d}}}{{\text{P}}_{\text{s}}-{\text{P}}_{\text{d}}}$$

Volumetric compliance (${\text{C}}_{\text{v}}$) was defined by normalizing compliance to diastolic volume as follows:

2 $${\text{C}}_{\text{V}} = \frac{\text{C}}{{\text{V}}_{\text{d}}}= \frac{{\text{V}}_{\text{s}}-{\text{V}}_{\text{d}}}{{\text{V}}_{\text{d}}{ \times (}{\text{P}}_{\text{s}}-{\text{P}}_{\text{d}}\text{)}} \times {100\% }\left[{\text{\% mmHg}}^{-1}\right]$$

To facilitate comparison with previously published clinical data by Pascaner et al., a pulse pressure with P_s_–P_d_ amounting to 56 mmHg (130–74 mmHg) was assumed to calculate ${\text{C}}_{\text{v}}$ based on Eq. (2) ^27^.

### Diameter distensibility

**Figure 5:**
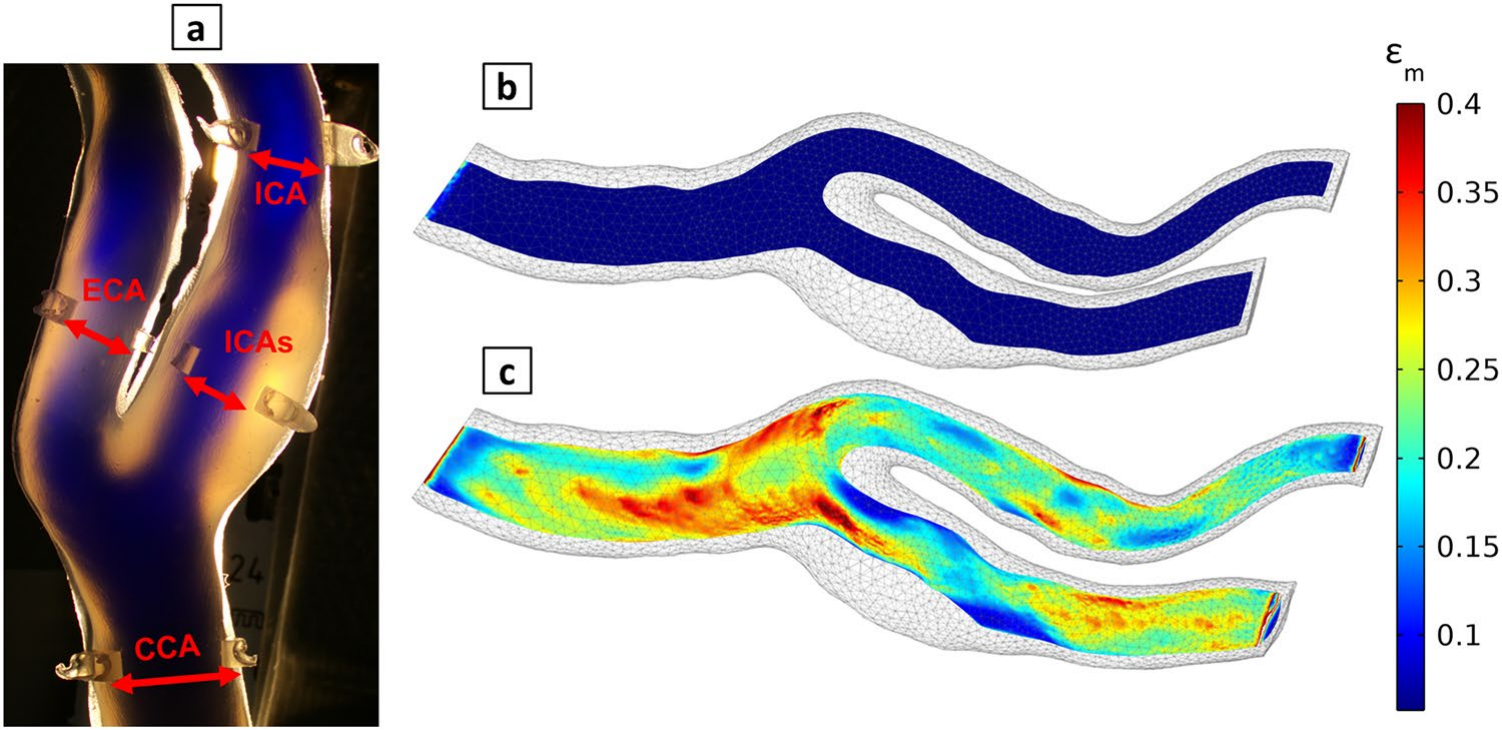
Experimental model with markers at four positions (CCA, ECA, ICA, and ICAs) for the measurement of diameter distensibility (**a**); strain distribution in the numerical model in the initial state (**b**) and at 140 mmHg (**c**).

The diameter distensibility (${\text{C}}_{\text{D}}$) is defined as the percentage ratio of the diameter increase relative to its initial diameter to the pressure increase:

3 $${\text{C}}_{\text{D}}=\frac{{\text{D}}_{\text{s}}-{\text{D}}_{\text{d}}}{{\text{D}}_{\text{d}} \times \text{(}{\text{P}}_{\text{s}}-{\text{P}}_{\text{d}}\text{)}} \times {100\% }\left[{\text{\% mmHg}}^{-1}\right]$$

where ${\text{D}}_{\text{s}}$ and ${\text{D}}_{\text{d}}$ are the vessel diameters at systole and diastole, respectively. During the volumetric compliance test, the samples were placed under the microscope, and some drops of ink were added to the water to enhance the visibility of the vessel lumen. The distance between both vessel walls was measured at the marker sites after each pumping step (Fig. 5a). Despite representing the projection of a noncircular cross-section, this distance was defined as the vessel's inner diameter for the calculation of diameter distensibility. To ensure consistent comparison with the numerical simulations, the “numerical” diameter at each marker position was defined by the projecting the vessel cross-section onto a plane resembling the laboratory experiment’s configuration plane, as depicted in Fig. 5a.

### Numerical simulation

The distal outflow regions of the ECA and ICA were sealed with a solid material structure over a length of approximately 1 mm in length in the numerical environment (Fig. 5). This configuration accounts for the closure of outflow branches in the experimental setup (Fig. 4), resulting in a fluid domain with only one inflow boundary. The time-dependent Navier–Stokes equations governing incompressible fluid motion and a linear elastic isotropic material model for the structural deformation of the vessel wall were employed in our numerical FSI model. The Arbitrary Lagrangian Eulerian (ALE) description approach was used to address the moving domain problem^28^.

The elastic modulus of the solid material was a key aspect of our investigations in the numerical compliance study. A linear elastic model was applied using both a constant elastic modulus (${\text{E}}_{\text{const}}$) and a E $({\varepsilon}_{\text{m}}$) based on the stress–strain measurements depicted in Fig. 8. The ${\varepsilon}_{\text{m}}=\frac{\gamma }{\sqrt{2}(1+\nu )}+ \frac{\left|{\varepsilon }_{vol}\right|}{3\left(1-2\nu \right)}$ was chosen to represent the strain dependency of elastic modulus in our simulations by combining the volumetric strain ${\varepsilon}_{\text{vol}}=\text{trace}({\varepsilon})$, which is the first invariant of the Green–Lagrange strain tensor describing the material compressibility, and the shear strain $\gamma =\sqrt{2 \text{dev}({\varepsilon):}\text{dev}({\varepsilon)}}$, which is related to the second invariant of the deviatoric strain $\text{dev}({\varepsilon)=\varepsilon-}\frac{1}{3}{\varepsilon}_{vol} \cdot {\text{I}}$, accounting for the shape change. The Poisson’s ratio $\nu$ was set to 0.4 to simulate nearly incompressible material behavior and avoid numerical instability associated with the critical value of 0.5 for incompressible material.

A constant inflow velocity profile was prescribed and its amplitude was adjusted to ensure that the volumetric flow produced a 100% fluid volume increase over the computation time. The velocity amplitude was multiplied by the time variable to mimic the inflow increase and, thus, the increase in the pressure inside the model. The inflow section of the CCA was constrained with zero wall deformation, whereas the rest of the structure was allowed to move freely according to the FSI mechanism at the shared fluid–structure interface. The continuity of the solid (wall) deformation velocity and fluid velocity, as well as the continuity of forces, were maintained by the FSI model, leading to a no-slip relative fluid velocity.

The fluid was modeled with a density of $\text{1} \times {10}^{3}$ kg m^-3^ and a viscosity of $\text{1} \times {10}^{-3}$ Pa s. The density of the solid material was $\text{1.07} \times {10}^{3}$ kg m^−3^, and both E_const_ = 0.31 MPa and the described E $({\varepsilon}_{\text{m}}$) functions were implemented based on the tensile measurements of both materials.

The FSI problem was solved using the finite-element-based commercial software COMSOL Multiphysics 5.6 (COMSOL Inc., Stockholm, Sweden) with its MEMS Module, applying a monolithic fully coupled approach for the fluid and structure problems and incorporating the ALE moving domain method. Linear P1-P1 elements were used for fluid discretization, and quadratic serendipity elements were employed for solid domain discretization.

A grid independence study was performed to validate our numerical setup. The convergence of the numerical solution was investigated for a sequence of seven mesh resolutions ranging from 27,000 (mesh no. 1) to 1.66 million (mesh no. 7) nonconformal (tetrahedral and prismatic) elements, resulting in an average mesh size ranging from 1.97 to 0.85 mm. The numerical error was calculated with respect to the high-resolution solution with the finest mesh no. 7 as well as by the difference between the numerical solutions for two consecutive meshes. Both approaches showed a clear decrease in various error types with increasing the number of elements. The relative error of vessel volume at a specific pressure of 120 mmHg decreased from 1.216% between mesh no. 1 and 2 to 0.125% for mesh no. 6 and 7. A similar decrease in relative error (from 1.76 to 0.15%) was observed for volumetric compliance at the same mesh settings. Our mesh convergence study confirmed that the numerical solution converges to a high-resolution reference solution and the difference in wall compliance indicators, such as vessel volume and pressure–volume curves, decreases with increasing mesh resolution.

The computational mesh used in this study, including boundary layers, consisted of 133,388 tetrahedral elements in the fluid domain and with 40,612 prisms in two boundary layers. The structure domain consisted of 106,105 tetrahedral elements. The total number of the elements in this computational mesh corresponds to a mesh size between mesh no. 4 and 5 in our convergence study, resulting in an expected relative change in vessel volume between 0.48% and 0.31%.

The implicit Euler method with adaptive time-stepping and a maximum step size of T/1000 was applied for time discretization, with a maximum of 20 iterations needed to achieve the convergence of the nonlinear Newton solver. The underlying linear systems were solved with the PARDISO solver.

### Feasibility of fluid dynamics assessment

**Figure 6:**
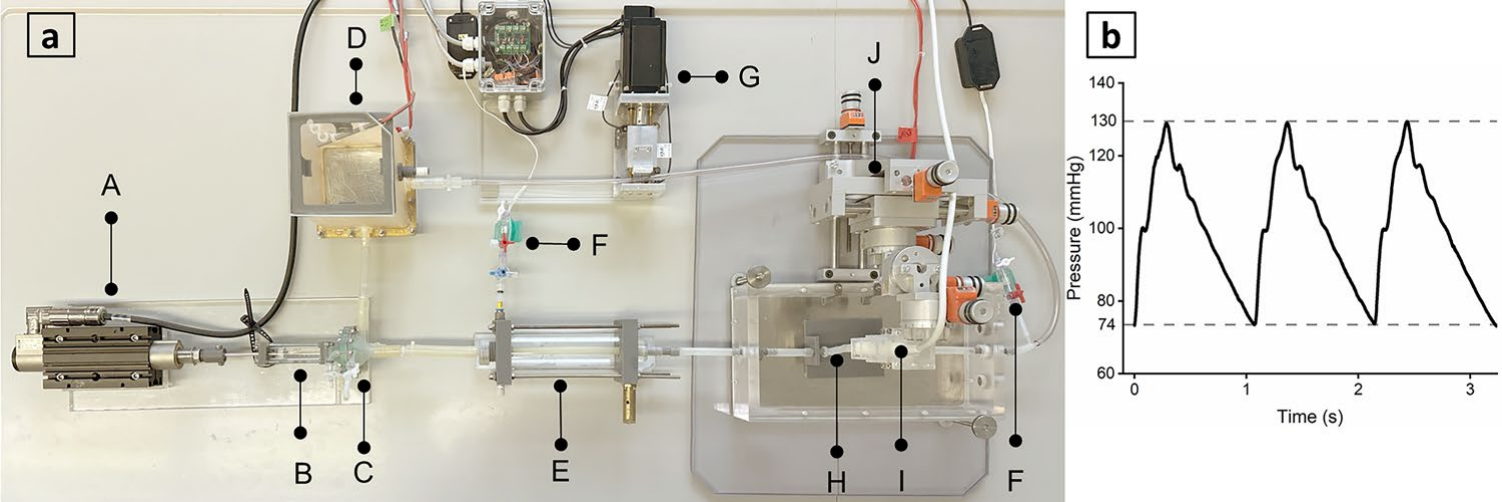
Pulsatile pump set-up (**a**): linear motor (A), syringe (B), pump chamber (C), reservoir (D), compliance chamber (E), pressure transducers (F), resistance (G), vessel model (H), ultrasound probe (I), ultrasound probe stabilizer (J); Pulse pressure (74–130 mmHg) waveform measured at the outlet of PVA-H model (**b**).

The PVA-H carotid bifurcation model was integrated into a mock circulatory loop, including an in-house pulsatile pump, a compliance chamber, resistance element, a reservoir, and tubes (Fig. 6a). The pump was constructed using a linear motor (NTI AG LinMot & MagSpring, Spreitenbach, Switzerland) and connected to a 10-ml syringe (SETonic GmbH, Ilmenau, Germany) and a 3D-printed pump chamber (Form 3, Formlabs Inc., MA, USA) for the displacement of test fluid. with a volumetric flow rate of 200 ml/min and a frequency of 55 bpm was connected to. Tests were conducted under the pulse pressure depicted in Fig. 6b and continuously recorded by a pressure transducer (Combitras, B. Braun SE, Germany) placed at the outlet of the vessel model. The averaged volumetric flow rate amounted to 200 ml/min at a frequency of 55 bpm.

A blood mimicking fluid (BMF) tailored to mimic blood viscosity (3.6–4 cP) was prepared according to European standard EN 61685, incorporating polyamide scattering particles (Orgasol™, ELF Atochem, France) with a diameter of 5 μm for ultrasound velocimetry. The viscosity was verified using an Ubbelohde viscometer (Thermo Fischer Scientific Inc., U.S.).

An ultrasound system (Lisendo 880, FujiFilm, Tokyo, Japan) equipped with vector flow mapping (VFM) function was used for evaluation of fluid dynamics on the longitudinal plane of the vessel model, encompassing CCA, ICA, and ECA. To evaluate the velocity vectors and WSS at specific points in the cardiac cycle, the pressure sensor voltage output (0–10 V) was adapted to the input voltage of the built-in ECG device of the ultrasound system (0–5 mV). The vessel model was subjected to pulsatile flow condition during the experiments, which lasted approximately 20 min.

### Statistics

The data were analyzed using Excel 16 (Microsoft, Washington, USA) and plots were generated using OriginPro 2022 (OriginLab Corporation, Massachusetts, USA). All the data are presented as the mean values ($\mu= \sum_{{\text{i}}= \text{1} }^{\text{n}}{{\text{x}}}_{\text{i}}/{\text{n}}$) and standard deviation (SD) ($\sigma = \sqrt{\sum_{\text{i=1}}^{\text{n}}{\text{(}{\text{x}}_{\text{i}}- \mu \text{)}}^{2}/{\text{n}}}$). In the x–y diagrams, the curves were averaged in OriginPro using linear interpolation over the full x range, and the error bars indicate the SDs.

The Shapiro–Wilk test was performed by SPSS 29 (IBM Corporation, New York, USA) to assess the normal distribution of the data. For normally distributed samples, differences between the groups were compared with two-sample t-test assuming unequal variances and the statistical significance of differences between each material and concrete values of numerical simulations was evaluated with one-sample t-test. Accordingly, non-normally distributed data were compared by Mann–Whitney test for two independent samples and Wilcoxon Signed-Rank test for a single sample in Excel utilizing “Real Statistics Resource Pack” software. Statistical significance was defined as a two-tailed p-value ≤ 0.05.

Linear regression analysis was performed on the pressure-diameter dataset of each measurement to derive a best-fitting line. Outliers outside three SD were identified and considered in the analysis, with their inclusion justified by their potential impact on the overall dataset.

## Results

### Geometric accuracy

The measured wall thicknesses of the CCA, ECA, and ICA in PVA-H and silicone samples (n = 5) as well as in the STL models of imaging data were averaged (Table 1). In the physical models, the average and SD were obtained over all samples, slices, and positions on the circumference, as shown in Fig. 3, while in the numerical model, the SD refers only to different slices and positions.

**Table 1:** The average wall thickness (± SD) of STL model, PVA-H, and silicone vessel models.

	CCA	ICA	ECA
STL model	1.59 ± 0.13 (mm)	1.10 ± 0.15 (mm)	1.13 ± 0.18 (mm)
PVA-H	1.36 ± 0.30 (mm)	0.92 ± 0.30 (mm)	0.92 ± 0.35 (mm)
Silicone	1.62 ± 0.26 (mm)	1.14 ± 0.27 (mm)	1.24 ± 0.38 (mm)

The average wall thickness of both PVA-H and silicone samples fell within the range of imaging data, with a greater percentage error in the PVA-H samples (16.9–22.8%) than in the silicone samples (1.9–8.9%). Comparing the differences of silicone and PVA-H with imaging data, no significant difference was observed in silicone (p > 0.05), although the difference in PVA-H was statistically significant (p ≤ 0.05), indicating that the decreased wall thickness in the PVA-H samples was presumably caused by water loss and shrinkage after model preparation.

**Figure 7:**
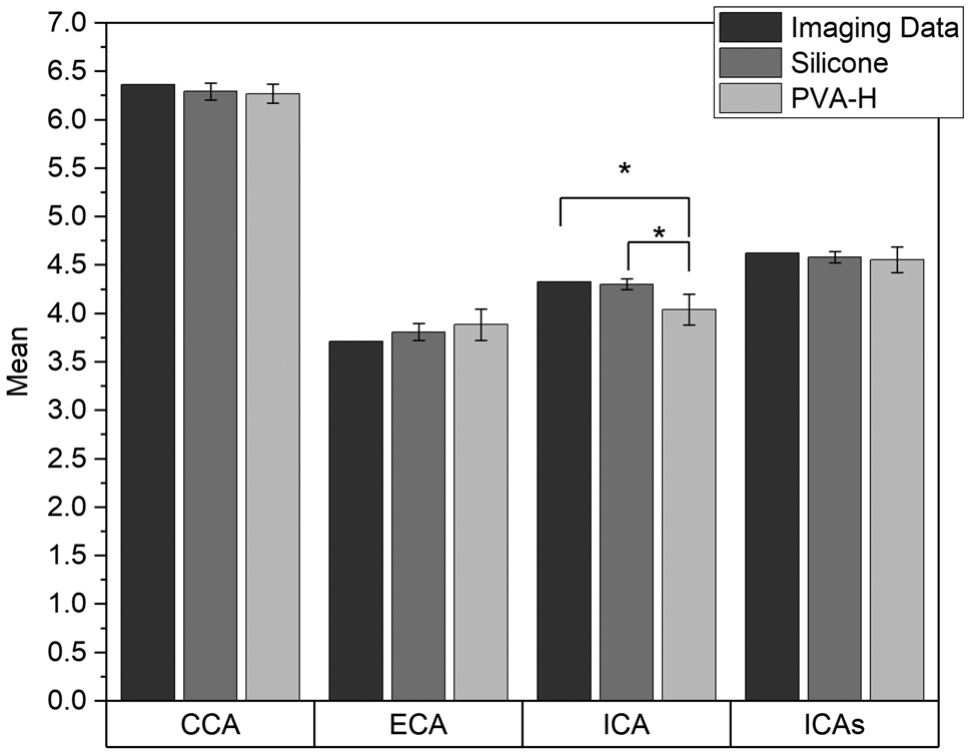
Effective diameter of the vessel lumen in four sections (CCA, ECA, ICA, ICAs) in imaging data compared to the average effective diameter of silicone and PVA-H models at corresponding marked positions (n = 5; ^∗^p ≤ 0.05).

Figure 7 displays the average effective diameter, calculated based on Eq. (1), at four sites: CCA, ICAs, ECA, and ICA for all models made of PVA-H and silicone as well as for STL models of imaging data used for computational geometry in numerical simulations. The maximum percentage error observed was 7% at the ICA position of the PVA-H samples compared to the STL model of imaging data. Statistical analysis revealed no significant difference (p > 0.05) among the different groups, except for PVA-H at the ICA site compared to both silicone and imaging data (p ≤ 0.05). This suggests that, overall, there were minor discrepancies in the effective diameter between the physical models and the imaging data, with the PVA-H samples exhibiting a slightly greater deviation at the ICA site.

### Elasticity

**Figure 8:**
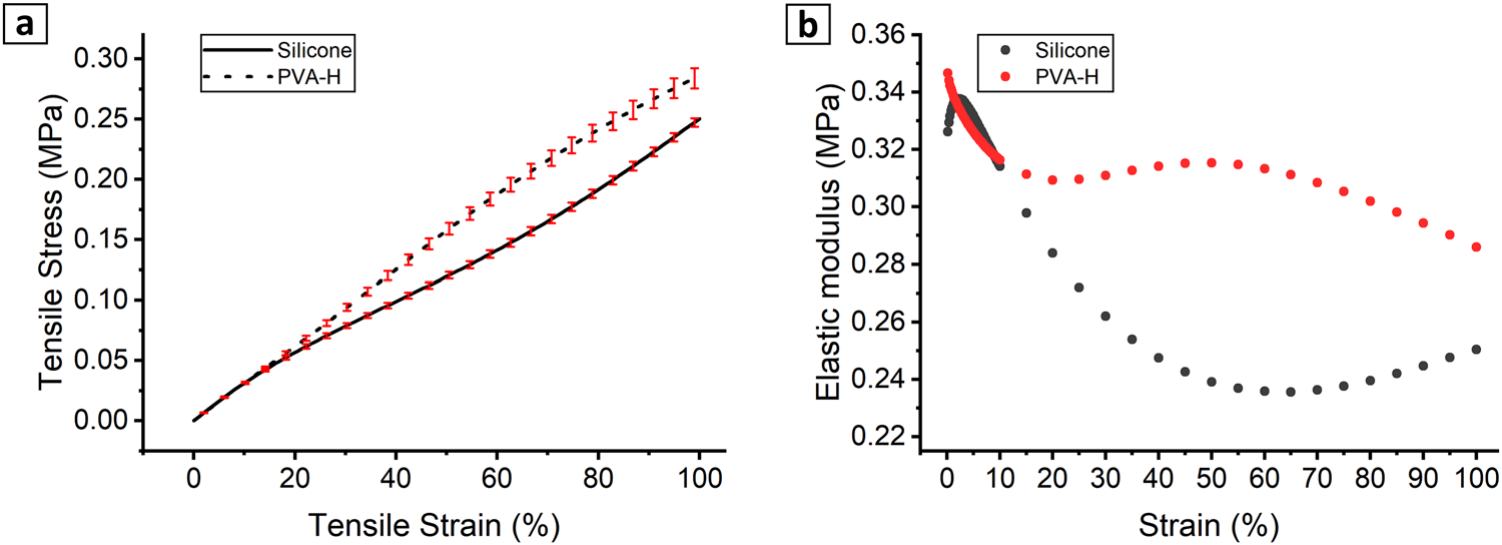
Stress–strain relationship of silicone and PVA-H averaged among the samples (**a**) and elastic modulus of silicone and PVA-H at different strain values (**b**).

Figure 8 illustrates the average curves of the stress–strain relationship for both silicone and PVA-H materials (n = 5), which were determined using uniaxial tensile tests. The curves show a noticeable drift from each other at higher strain values. Despite having nearly similar elastic moduli within the low strain range (0–20%), amounting to 0.31 ± 0.007 MPa for PVA-H and 0.29 ± 0.007 MPa for silicone, these values are significantly different (p ≤ 0.05) between the two materials. Nevertheless, it is worth noting that these values fall within the lower range of the vascular tissue elastic modulus of 0.2–0.6 MPa under physiological pressure^29^.

The elasticity function $\text{E (}{\varepsilon}_{\text{m}}\text{)}$ used in the numerical simulations, derived as the piecewise slope of the measured stress–strain curve (Fig. 8b), shows strongly nonconstant behavior with respect to strain, even within small strain intervals < 10%, which affects volumetric compliance and diameter distensibility. In Fig. 5, the surface distribution of the chosen ${\varepsilon}_{\text{m}},$ consisting of strain invariants as shear and volumetric strains can be observed in the numerical model, which causes spatial variation in elastic modulus in our simulations due to the strain dependency of elastic modulus, especially for silicone material as shown in Fig. 8b.

### Volumetric compliance

**Figure 9:**
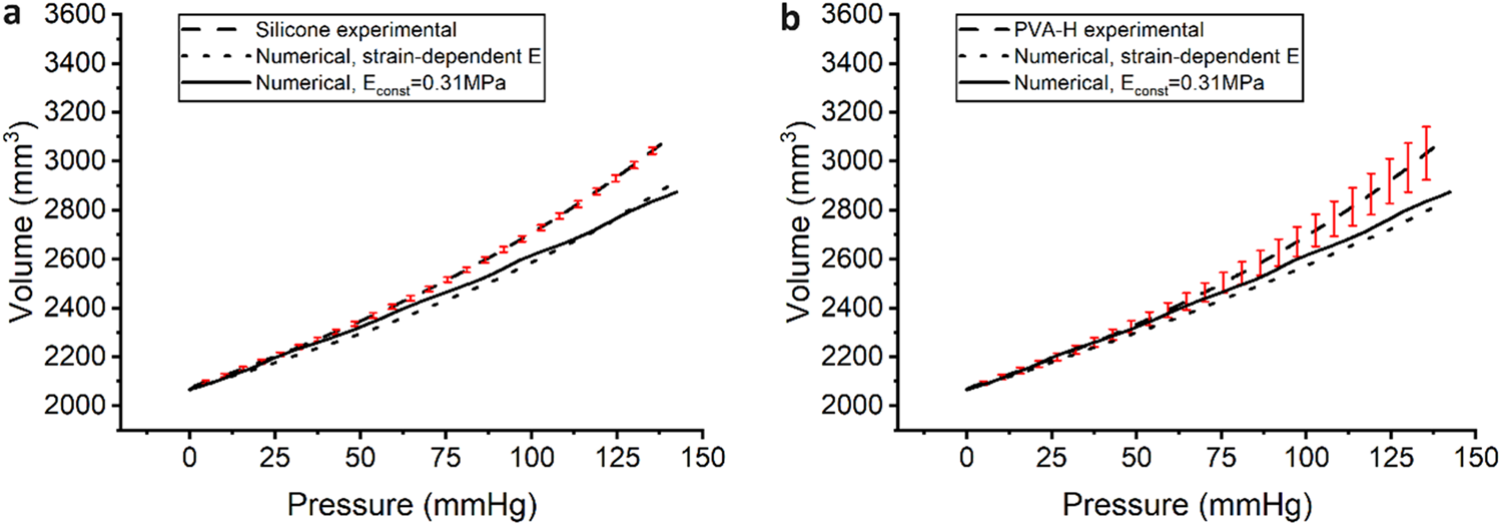
Experimental and numerical pressure–volume relationships of the silicone (**a**) and PVA-H (**b**) vessel models with error bars indicating the SDs of the experimental measurements. The numerical P–V curves are represented for both $\text{E (}{\varepsilon}_{\text{m}}\text{)}$ and E_const_.

Figure 9 displays the pressure–volume relationship for all the samples, with the average curve and SD depicted for both materials. The PVA-H samples showed a greater deviation from the average compared to silicone samples. For comparison, numerical pressure–volume curves were obtained by implementing a linear elasticity model with a E_const_ of 0.31 MPa for both materials, as well as an elasticity model using $\text{E (}{\varepsilon}_{\text{m}}\text{)}$ derived from tensile tests, as described above and presented in Fig. 8.

The value of E_const_ was obtained as the slope of the linear fit to the average stress–strain curve (Fig. 8a) in the strain interval (0,${\varepsilon}_{\text{max}}$), where ${\varepsilon}_{\text{max}}{=20\%}$ is the surface average of the ${\varepsilon}_{\text{m}}$ reached at a pressure of 140 mmHg, assuming the maximum physiological pressure. Since the stress–strain curves for both materials overlap in this strain range, a similar value of E_const_ = 0.31 MPa was obtained for PVA-H and silicone.

Table 2 presents the volumetric compliance calculated from all the measurements, with the overall mean and SD depicted across the five specimens. The volumetric compliance in the numerical simulations was lower than that in the experimental values. Moreover, the experimentally determined values were nearly identical for both materials. However, in numerical modeling with an averaged E_const_ = 0.31 MPa, the volumetric compliance is lower than that obtained using $\text{E (}{\varepsilon}_{\text{m}}\text{)}$ function for silicone. These results were in accordance with the behavior of the pressure–volume curves in Fig. 9, where lower volume increases were observed in numerical simulations. The slope of the pressure–volume curve was even lower for PVA-H than for the other samples using $\text{E (}{\varepsilon}_{\text{m}}\text{)}$ function. The overlap of the numerical curves for PVA-H, whether using a E_const_ or a $\text{E (}{\varepsilon}_{\text{m}}\text{)}$ function, can be attributed to the behavior of its elasticity function (Fig. 8b), in which the values of $\text{E (}{\varepsilon}_{\text{m}}\text{)}$ vary less within the realized strain range (locally up to 40% strain even at a high pressure of 140 mmHg as seen in Fig. 5). This variation results in an average value of 0.312 MPa within this range, which is similar to the chosen constant value of 0.310 MPa. In contrast, the larger decrease in the $\text{E (}{\varepsilon}_{\text{m}}\text{)}$ curve for silicone within the strain range of 10–40% results in higher values of silicone volumetric compliance. Overall, the statistical analysis revealed that the difference between the silicone and PVA-H samples was not significant (p > 0.05), while a significant difference from the numerical simulation was observed (p ≤ 0.05).

**Table 2:** Volumetric compliance values at clinical pulse pressure (74-130 mmHg) for PVA-H, silicone, and E_const_.

	Volumetric compliance (± SD) [% mmHg^−1^]	
	Experimental	Numerical
PVA-H	0.346 (± 0.040)	0.248
Silicone	0.342 (± 0.004)	0.290
E_const_	NA	0.255

### Diameter distensibility

**Figure 10:**
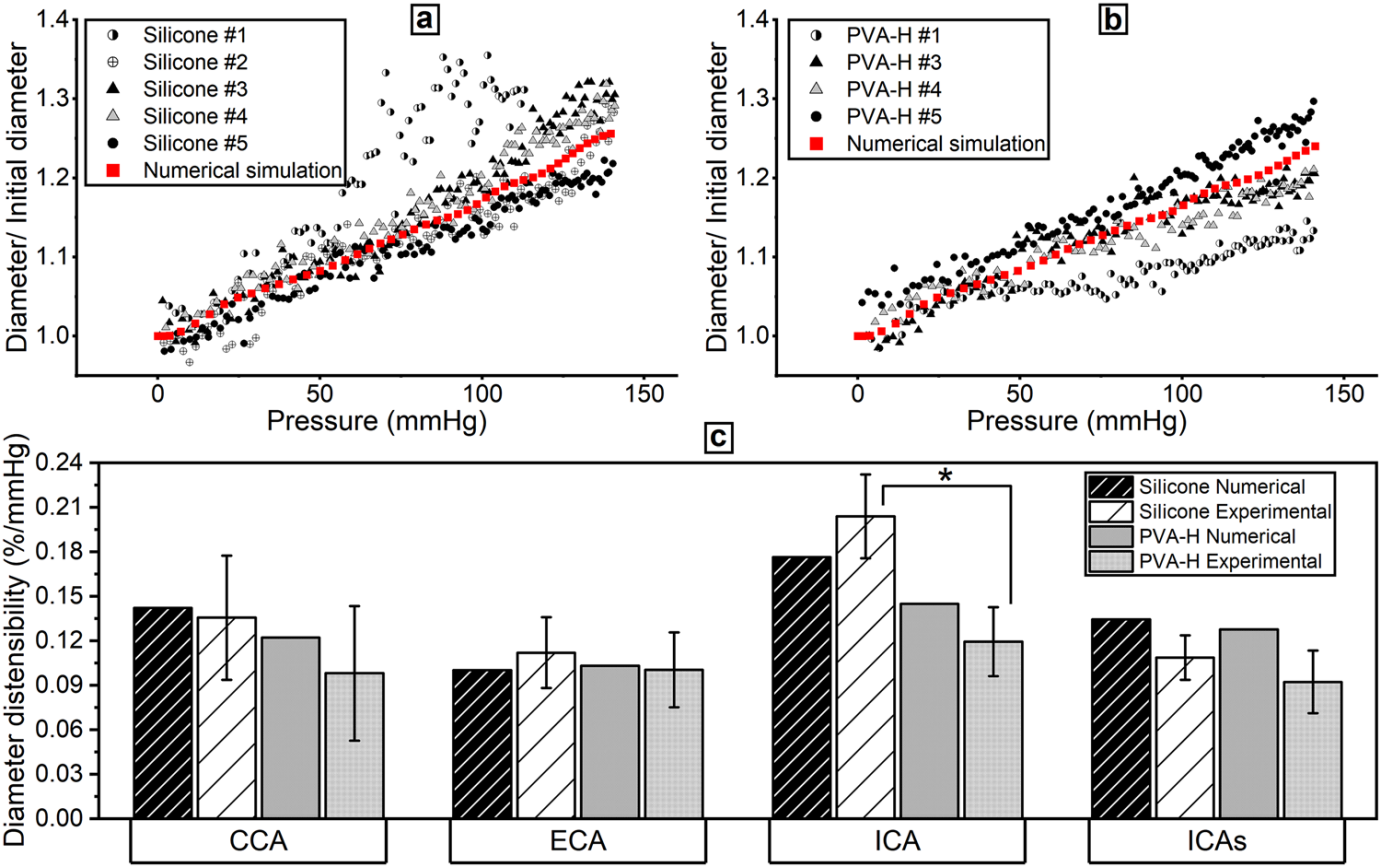
Experimental and numerical diameter-pressure relationship at ICA site in five silicone (**a**) and four PVA-H samples (**b**); experimental and numerical diameter distensibilities at different vessel sites (CCA, ECA, ICA, and ICAs).

The diameter change resulting from the pressure increase was measured and averaged across all the samples at different locations for both materials. Using the ICA as a representative location, Fig. 10 depicts these measurements and numerical curves for both silicone (a) and PVA-H (b). The diameter distensibility was calculated within a physiological pressure range of 74–130 mmHg (Fig. 10c). Comparing silicone and PVA-H samples, there was no significant difference observed except for the ICA (p ≤ 0.05). In the silicone samples, the experimental diameter distensibility ranged from 0.11% mmHg^−1^ in the stenotic ICA region to 0.20% mmHg^−1^ in the healthy ICA region. In the PVA-H models, the estimated experimental values were in a narrower range, amounting to 0.09–0.12% mmHg^−1^, with the minimum and maximum detected in the stenotic and healthy regions of the ICA, respectively.

Overall, comparing the experimental and numerical values did not provide a clear trend; however, almost all the obtained numerical distensibilities fell within the range of measuring errors of the experimental values. A significant difference (p ≤ 0.05) between the experimental and numerical results occurred in the stenosed ICA for both material groups, with maximal discrepancies amounting to 0.026 and 0.036% mmHg^−1^ as the absolute values for silicone and PVA-H, respectively; these values are greater than the measuring error.

### Feasibility of fluid dynamics assessment

**Figure 11:**
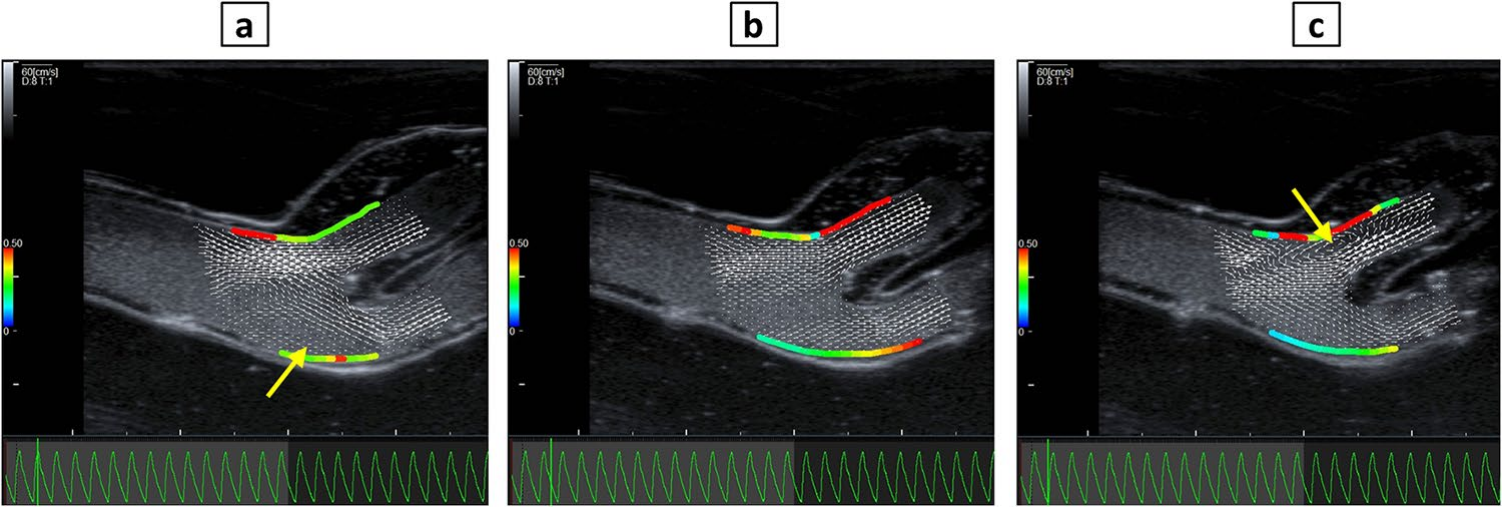
Vector velocities measured by VFM at peak systolic pressure (**a**), diastole (**b**), and end of diastole (**c**) with arrows showing the recirculation zones. WSS values are depicted on the walls (color bar ranging 0–0.6 Pa).

The velocity vectors at PVA-H carotid artery bifurcation during 15 pulsation cycles are depicted in Supplementary Video S1. Three frames of this video are selected and shown in Fig. 11. The Fig. 11a demonstrates the velocity vectors at peak systolic pressure, depicting a recirculation zone at carotid bulb. A second recirculation zone occurred at the end of diastole, shortly before the beginning of the systolic phase at the carotid bulb proximal to ICA, as shown in Fig. 11c. The absolute values of WSS over the all cycle ranged between 0.1 and 0.6 Pa on the outer wall of ICA adjacent to stenosis, with the maximum WSS occurring during diastole presumably due to pressure-flow waveform shift as previously shown^30^.

## Discussion

In this study, our objective was to fabricate and characterize thin-wall, compliant carotid bifurcation models made from PVA-H to be used for in vitro investigations of fluid dynamics and vascular implant mechanics. PVA-H, a material with promising properties for cardiovascular research due to high transparency and tunable mechanical properties, exhibited similar low-strain elastic modulus and volumetric compliance to natural vessels. Wall thickness and effective diameter of the vessel models were measured, demonstrating the capability of our fabrication method to manufacture thin-walled anatomical models with a minimum thickness of approximately 1 mm. For comparison, models were also produced using silicone material. Additionally, a numerical model was developed to complement the experiments as a potential auxiliary tool for predicting the influence of model geometry and material on compliance, thus supporting and directing the fabrication of physical vessel models. A PVA-H model was integrated into an in-house mock circulatory loop with semi-physiological pulse pressure, demonstrating the practical application of the model in ultrasound-based investigation of fluid dynamic at carotid artery bifurcation.

In recent decades, in vitro vascular models, primarily made from silicone, have been fabricated to replicate the anatomical^9,18^, mechanical^31,32^, and biological^33,34^ properties of natural vessels. Therefore, several patient-specific vascular models have been created to study flow characteristics in diseased vessel regions, such as stenosis and aneurysms, and to evaluate the effects of their treatments by means of catheter systems and implants^9,35^. However, in none of the mentioned studies, thin-wall anatomical models made of PVA-H were characterized in terms of their mechanical properties, considering volumetric compliance and local distensibility, which are crucial factors influencing both fluid dynamics and implant behavior. Clauser et al. assessed the effect of intracranial braided stents (flow diverters) on flow patterns in an aneurysm model. In this work, the vessel elasticity was neglected by employing a nearly rigid silicone block, which was introduced as a limitation in capturing the dynamics of complex flow patterns as stated by Clauser et al.^13^. Similarly, in the study conducted by Ding et al. vessel elasticity was neglected in the investigation of flow patterns affected by a flow diverter within a nearly rigid silicone model^18^. Furthermore, McCulloch et al. described that in vitro assessment of implants through endothelialization of in vitro models could be enhanced by incorporating more complex geometries with proper mechanics^34^. In this regard, our study proposed a combination of numerical and experimental tools for the fabricating and characterizing a new generation of thin-wall vascular models made of PVA-H, a promising material for cardiovascular research in terms of transparency^36^, friction^37^, cell compatibility^20–23^, and thus potential endothelialization. Finally, we compared PVA-H model with silicone model of similar elasticity and integrated a PVA-H model into a mock circulatory loop for fluid dynamics assessment.

The improvement of stroke treatment as well as prevention strategies requires a thorough understanding of the hemodynamics and the effects of endovascular interventions on fluid dynamics at the carotid bifurcation. The practical application of ultrasound-based investigation of fluid dynamics within PVA-H vessel integrated into a pulsatile pump was demonstrated in this study. Furthermore, the PVA-H with high light transmission (99.8 ± 0.2%)^36^ and required index of refraction (˂ 1.55)^38^ is potentially suitable for use in particle image velocimetry (PIV) as well. Moreover, based on our experience, transparent PVA-H models enable the visualization of implant deformation using high-frame-rate microscopy cameras under pulsation.

The influence of a compliant standardized stenosis model made of PVA-H compared to a rigid model made of silicone on the flow condition was proven^16^. In another study by Poepping et al., flow and turbulence in semicompliant and rigid carotid models made of polydimethylsiloxane were investigated. The results revealed different flow patterns and turbulence intensities in the two models due to the different compliance^41^. Simplified rigid and compliant artery models of healthy CCAs were compared using PIV. The results demonstrated a significantly overestimated WSS in the rigid model^42^. Therefore, these studies emphasize the importance of vascular models with physiological compliance for realistic fluid dynamic investigations and implant characterization^41^. However, in these works, neither the volumetric compliance nor the diameter distensibility was characterized.

Vessel compliance is influenced by geometrical properties such as vessel diameter and wall thickness, as well as mechanical properties of the vessel wall, which are generally described considering the vessel as a continuum and defining an average elastic modulus. Ebrahimi et al. reported the elastic modulus of actual cerebral arteries to range between 0.2 and 0.6 MPa in the physiological pressure range, depending on the microstructure of the vessel wall at various anatomical locations^29^. The elastic modulus of CCA in 19 patients was obtained by measuring the diameter during the systolic and diastolic phases using pixel tracing of successive frames and blood pressure in B-mode ultrasound, amounting to as 0.453 ± 0.099 MPa^42^. In our study, the elastic modulus was 0.31 ± 0.008 MPa and 0.29 ± 0.007 MPa for PVA-H and silicone, respectively, and thus lying in the lower range presented in the literature. Nevertheless, the mechanics of the PVA-H is tunable^43^ and this possibility is limited while working with silicones. Preparation of PVA-H using PVA with higher molecular weight^43^ and higher concentrations^21^, as well as an increase in the number of freeze–thaw cycles^44^ could rise the elastic modulus and thus decrease the vessel compliance.

Volumetric and cross-sectional distensibilities of carotid arteries in 19 patients with a pulse pressure of 56 ± 9 mmHg (74–130 mmHg) were measured using B-mode ultrasound sequences and resulted in 0.419 ± 0.228 and 0.342 ± 0.115% mmHg^−1^, respectively^27^. The arterial volumetric compliance of the cerebral arteries originating from the circle of Willis in five healthy volunteers was measured using arterial spin labeling in magnetic resonance imaging; the results ranged from 0.4% to 1.1% mmHg^−1^^45^. In our study, variable wall thickness according to post-processing of medical imaging combined with elastic modulus in a physiological range led to experimental volumetric compliance values of 0.346 and 0.342% mmHg^−1^ in the pressure range of 74–130 mmHg for PVA-H and silicone, respectively. Accordingly, these results lay on the lower limit of actual vascular mechanics ranges. The numerical volumetric compliance obtained by applying a generalized linear elasticity model with $\text{E (}{\varepsilon}_{\text{m}}\text{)}$ was generally lower (0.248 and 0.290% mmHg^−1^). However, compared to the numerical volumetric compliance obtained by the purely linear elastic model with E_const_, our adaptation of the elasticity model dependent on the chosen ${\varepsilon}_{\text{m}}$ provided a better match to the experiments.

While volumetric compliance is related to an integrated volume change over the whole vessel, diameter distensibility refers to local vessel deformation in defined segments. We consider the latter parameter to be of paramount importance in investigating the local effects of fluid dynamics and implant behavior. In our study, we defined four different regions in the CCA, ICA, ICAs, and ECA marked in the models for accurate correlation with the numerical model. The lumen change was measured at different pressures considering a single projection, with values ranging between 0.20 mm and 0.52 mm and a percentage change between 0.09 and 0.2% mmHg^−1^. Additionally, a good correlation with the numerical model was observed. In clinical studies using mainly ultrasound, the diameter distensibility of the carotid artery was measured in a wide range between 0.07 and 0.5% mmHg^−1^^27,46–49^.

The feasibility of ultrasound-based in vitro assessment of fluid dynamics under pulsatile flow was demonstrated in this study. In a previous clinical study, fluid dynamics at carotid bifurcation of eight volunteers was assessed in longitudinal plane with vector flow ultrasound^50^. A vortex with complex flow in carotid bulb in all the volunteers in different phases of the cardiac cycle was shown, comparable to the qualitative assessment of flow vectors performed in our study. The range of WSS values observed in PVA-H model is consistent with findings from a clinical study involving 20 patients, where WSS was indicated as 0.72 ± 0.30 Pa in ICAs^51^. In our investigation, we found that the peak wall shear stress (WSS) in ICAs did not coincide with the peak systolic pressure. This observation is also consistent with the findings of Wang et al., who highlighted the significance of the phase shift between pressure and WSS and proposed it to play a crucial role in identifying areas prone to atherosclerosis within the carotid artery^30^.

Our study presents several limitations. First, our preliminary study was performed using a single anatomical model to demonstrate the feasibility of fabricating a thin-wall vessel model made of PVA-H with mechanics similar to those of natural vessels. To enable a more comprehensive comparison with the numerical model and to better understand the relationships between materials, geometrical parameters, and mechanical parameters, further anatomies should be included in future studies.

Moreover, although both PVA-H and silicone demonstrated nonlinear stress–strain behavior in the experimental tensile strength curve, we implemented a variable (strain-dependent) elastic modulus into the numerical simulation based on a linear elastic material model. To replicate this nonlinear behavior, we generalized the linear elasticity model by adding an appropriate dependency of elastic modulus on strain, derived from tensile test measurements. While our numerical model with $\text{E (}{\varepsilon}_{\text{m}}\text{)}$ yielded improved values for the volumetric compliance of silicone, the response to the $\text{E (}{\varepsilon}_{\text{m}}\text{)}$ was low in the case of the PVA-H. This discrepancy might be due to tiny leaks in the PVA-H samples during the compliance experiments, potentially resulting in higher compliance values.

Although the vascular models fabricated in this study exhibited similar low-strain elastic modulus and volumetric compliance to natural vessels, the stress–strain curves of both materials differ from a healthy blood vessel. Blood vessels exhibit distinctive mechanical characteristics such as viscoelasticity, vasoconstriction or vasodilatation, anisotropy, and non-linear stress–strain behavior^52^, mainly resulting from the composition of blood vessels and the geometric arrangement of elastin and collagen fibers. Additionally, the viscoelastic properties of PVA-H, as demonstrated in previous studies^8^, were neither measured in the physical models, where tests were performed under nearly static conditions, nor considered in the numerical simulation. Furthermore, the presence of soft and calcified areas in the stenotic region was not implemented in the model, which was deemed homogeneous and isotropic. Developing a more comprehensive model that addresses viscoelastic properties, heterogeneity, and anisotropy would further enhance compliance prediction using both numerical and physical methods.

The static measurement of volumetric compliance and diameter distensibility in our in-vitro setup was performed with the vessel ends closed, which does not accurately represent realistic physiological conditions where vessels could elongate in the axial direction. This setup also differed from the numerical simulation, which allowed free deformation except for the inflow section. Therefore, dynamic measurement of volumetric compliance and diameter distensibility should be performed in a more complex setup under pulsatile conditions.

Local vessel distensibility was defined as the percentage change in diameter as the pressure increased. However, we measured the distance between two points (diameter) at a single projection under the microscope. In the future, despite higher complexity, medical ultrasound could provide better insight into the diameter distensibility by measuring the cross-sectional diameter of the artery in diastole and systole.

Achieving a leakage-free connection of PVA-H models to the pump during the tests posed a challenge due to the low friction and slippery surface of the PVA-H. Although efforts were made to ensure a secure connection, tiny leakages were observed in two samples at higher pressure values. These leakages were incidental and attributed to bubble formation during stirring process and material injection. Moreover, since the PVA-H models lose water and shrink over time, the experiments should be performed shortly after fabrication to minimize the deviations in the results among the samples. Efforts should be made to enhance PVA-H stability over time to increase the practicality of the material for in vitro experiments.

A feasibility of ultrasound-based fluid dynamic investigation was presented in this study. In this scope, the flow profile was analyzed primarily qualitative, and shear stress measurement were restricted to single regions of interest along the vessel walls. The validation of this method with reference to state-of-the-art optical techniques like PIV, especially across a broader range of fluid dynamic parameters and anatomies, is essential in future studies. Following the validation, the incorporation of the biological fluid such as the whole blood or blood-based products could provide insights into phenomena like thrombus formation in stenotic regions and the performance of implants in cardiovascular research.

## Conclusions

In conclusion, this study presents a novel method for fabricating and characterizing a vascular model with physiological compliance using PVA-H, which represent a promising material for cardiovascular research due to their tunable mechanics, low surface friction, and superior transparency. In terms of methods, our combined numerical and experimental approach is highly meaningful for predicting vessel model mechanics before fabrication, offering insights into material behavior and aiding in model optimization.

As a future perspective, the use of hydrogel might potentially allow for in-vitro model biologization in cardiovascular research. With favorable mechanical and physical characteristics assessed in this paper, and potential biocompatibility according to the literature, could lead the development of a more realistic and physiological environment for in vitro investigation of fluid dynamics and implant mechanics at carotid bifurcation in the future, thus, reducing the reliance on animal experiments and accelerating design developments.

### Supplementary Information

Supplementary Video 1.

## Data Availability

The data that support the findings of this study are openly available in “figshare” repository at 10.6084/m9.figshare.25041512.
